# Adolescents’ Knowledge on Climate Change: A Nationwide Study in Indonesia

**DOI:** 10.3390/ijerph22040571

**Published:** 2025-04-05

**Authors:** Evi Martha, Besral, Ulfi Hida Zainita, Naurah Assyifa Rilfi, Syifa Aulia Aminudin

**Affiliations:** 1 Department of Health Education and Behavioral Sciences, Faculty of Public Health, Universitas Indonesia, Building D 1st Floor, Depok 16424, Indonesia; ulfi.hida@ui.ac.id (U.H.Z.); naurah.assyifa@alumni.ui.ac.id (N.A.R.); syifa.aulia03@ui.ac.id (S.A.A.); 2 Department of Biostatistics and Population Studies, Faculty of Public Health, Universitas Indonesia, Building A 2nd Floor, Depok 16424, Indonesia

**Keywords:** climate change, knowledge, adolescent, health, well-being

## Abstract

Adolescents’ knowledge about climate change is key to protecting the well-being of all generations and to promoting individuals’ rights and resilience. This study assesses the climate change literacy of Indonesian adolescents and its determinants. This nationwide study was conducted in 2023 in Sumatra, Java, Kalimantan, Sulawesi, and Eastern Indonesia. A total of 1126 adolescents aged 13–19 years were selected through multi-stage sampling. The data were analyzed using the chi-square test and multinomial logistic regression. This study found that 49.7% of adolescents had poor climate change literacy. In the multivariate analysis, the significantly related factors had an odds ratio of 1.66–4.75. Climate change literacy was higher in adolescents from the West and Central Regions, from public or religious schools, and those with educated parents, than in adolescents from the Eastern Region, from private or vocational schools, and those whose parents had low education, respectively. This study suggests the need to promote equality in climate change literacy among Indonesian adolescents through formal and informal education. High-quality formal education would necessitate well-trained teachers with expertise in climate change, as well as a structured, age-appropriate curriculum. Meanwhile, informal education through another information dissemination and social media-based movements can help broaden outreach among adolescents.

## 1. Introduction

Climate change disrupts the well-being of individuals of all ages worldwide [1]. Climate change affects the ecosystems by altering climate patterns and causing ecosystem fragmentation [2]. Climate change intersects with several vulnerabilities of adolescents at varying risk levels because of various factors, including geography, poverty, gender, ethnicity, disability, chronic diseases, and the refugee status of natives [3,4,5].

The world has seen extreme changes in climate patterns recently, and this trend is predicted to greatly impact the already increased risk for 1.3 billion young people in 2050 [6]. Known as the largest archipelago country in the world, Indonesia is one of the countries that is vulnerable to climate change due to its subtropical climate and geographical location.

Ninety two percent of the 2342 disasters that occurred in 2016 were hydrometeorological in nature, with floods, landslides, and light tornados accounting for the majority of these events [3]. The regions of West Java, Central Java, East Java, Aceh, West Sumatra, North Sumatra, South Sumatra, and South Sulawesi saw the greatest numbers of floods between 2005 and 2018. In the meantime, landslides frequently happened in West Java, Central Java, East Java, West Sumatra, and Papua, potentially affecting up to 14 million people [2]. It is anticipated that the population exposed to these risks will grow in the absence of appropriate adaptation. For instance, Indonesia’s population at risk of a catastrophic river flood might increase by 1.4 million people by 2035–2044 if appropriate adaptation is not implemented [1]. The Indonesia National Disaster Management Agency reported that climate change has been proven to increase the frequency of disasters very drastically. There is a significant trend of natural disasters in Indonesia which has increased by 82% (2010–2022), especially through hydrometeorological disasters caused by climate change including wild fires, floods, droughts, landslides, storms, and typhoons [7].

Currently, there are 1.2 billion adolescents aged 10–19 years old worldwide, constituting 16% of the world’s population [8]. In Indonesia, the total population of adolescents aged 10–19 years in 2019 was 45,351,034, or 17.4% of the total population of Indonesia, the world’s fourth most populous country with over 260 million people [9].

Adolescent mortality is largely driven by environmental factors, such as unintentional injuries; physiological development renders adolescents more vulnerable to hazards, such as pollution, chemicals, poor urban health conditions, and climate change [8]. Parks et al. [10] found that an anomalously warm temperature higher by 1.5 °C for a year could significantly increase cases of injury-related deaths, with over 80% occurring in males, primarily from adolescence to middle age, highlighting the heightened risk adolescents face from severe climate events, including heat-related drowning and intentional harm such as assault or suicide. The WHO [11] reported that paying attention to the welfare of this subpopulation is crucial, as they are also in a critical period of emotional and social development, making them more vulnerable to the lasting adverse effects of climate change. Vicedo-Cabrera et al. [12] found that 37% of heat-related deaths may be attributed to anthropogenic (human-induced) climate change, and that increased mortality is evident on every continent, with the highest rates in Central and South America and Southeast Asia.

As environmental changes drive shifts in disease patterns, forced migration, food and water insecurity, and the rising number of climate refugees, several studies have examined the impact of climate-related factors on public health in Southeast Asia. Tropical diseases, in particular, have shown significant surges in response to changing climatic conditions, affecting millions across the region [13]. The results of a study in Laos found the relationship between climate factors and diarrhea cases, explained by the increase in rainfall causing river discharge to be higher so that the suspended sediment load became greater; this increases the cadre of fecal indicator bacteria, which causes an increase in diarrhea cases. Meanwhile, during the dry season, due to water shortages, people are forced to depend on contaminated water sources, thus worsening the spread of diarrheal disease [14].

The incidence of respiratory viruses in Vietnam is influenced by climate fluctuation. Among the most commonly found diseases are *influenza A viruses*, *rhinoviruses*, and *enteroviruses* (EVs). Their higher occurrence during the rainy season suggests a seasonal transmission pattern. Additionally, the prevalence of these viruses varies across time and space, highlighting the part that geography and the environment play in their dissemination [15]. There is a strong correlation between temperature, humidity, and rainfall patterns and malaria cases in Thailand. Rainfall in the same month has been linked to higher rates of *P. vivax* and overall malaria cases. Multiple environmental factors contribute to the transmission of malaria, as seen by the poor link found between forest cover and malaria incidence at the sub-district level [16].

Beyond infectious diseases, climate change is also exacerbating mental health challenges in the region. In Indonesia, climate change exacerbates mental health issues, including anxiety, depression, and post-traumatic stress disorder, among adolescents [17,18]. Qinanthi et al. [19] found that exposure to climate change-related events, such as extreme weather, can lead to significant psychological distress among Indonesian students, affecting their overall mental well-being [19]. Extreme weather events linked to climate change contribute to rising levels of anxiety, depression, and post-traumatic stress disorder (PTSD), particularly among adolescents. Another study found that exposure to climate-related disasters significantly affects the psychological well-being of Indonesian students, highlighting the broader mental health burden induced by environmental instability. Given these widespread and growing health impacts, Southeast Asia—home to over 600 million people—has been identified as one of the most climate-vulnerable regions in the world [19].

Adolescents are at a critical point in their biological, emotional, and social development, many of aspects of which will determine their lifelong well-being [20]. According to Ross et al., the five domains for adolescents’ well-being are as follows: good health and optimal nutrition; connectedness, positive values, and contribution to society; positive safety and environment; learning, competence, education, skills, and work eligibility; and institutions and resilience [20]. Climate change poses risks to these five domains and threatens the basic rights of children and adolescents [21]. Adolescents are our future leaders and agents of change who will make decisions in the face of increasingly severe climate conditions. Actions that mitigate climate change are important in protecting the current generation of adolescents and future ones [11]. However, in Indonesia, studies assessing adolescents’ knowledge about climate change remain limited. Some studies in Indonesia found that adolescents’ knowledge of climate change remains poor [22,23,24,25]. In fact, adolescents’ knowledge about climate change is one of the factors that must be improved so that adolescents can take actions that mitigate climate change and achieve good health and well-being for themselves and for future generations. This endeavor has become even more urgent, and one report highlights that young people in Indonesia are among those at the highest risk of experiencing the impacts of climate change, threatening their health, education, and well-being [26].

Meanwhile, knowledge about climate change is closely related to sources of information. School subjects are the main sources of information about climate change for 80% of adolescents in Indonesia [23]. However, several studies found that knowledge of climate change among Indonesian adolescents remains poor. This shows suboptimal climate change literacy in schools, which must be supported by government policies. The Ministry of Education and Culture of Indonesia has not expressed support for the sixth development agenda, namely, building the environment, increasing disaster resilience and climate change, in the Amendment of the Minister of Education and Culture Regulation Number 13 of 2022 [27]. This shows that the Ministry of Education and Culture has not been involved in overseeing climate change literacy in schools.

Apart from formal education, other sources of information from which adolescents gain climate change literacy. Study among Indonesia’s Generation Z found that social media campaigns can be an effective way to increase awareness about climate change, because the campaign has a positive impact on their attitudes and behavior towards the issue [28]. A 2020 report showed that, in Indonesia, adolescents constitute the largest group of internet users; they use the internet to learn via various platforms, including social media [29]. Internet usage increased especially during the COVID-19 pandemic, as Indonesian adolescents had to take classes through distance learning. During the post-pandemic transition in 2023, adolescents in Indonesia have already become accustomed to using technology and the internet in nearly all aspects of their lives.

It is important to recognize that knowledge of climate change is a foundation for competency development that leads to adjustments in one’s actions and behavior in relation to the environment [25]. Good knowledge and credible information about climate change can be valuable investments in reducing the risks of climate change, which threatens the rights of Indonesian adolescents. On the basis of this background, this study aims to assess Indonesian adolescents’ knowledge about climate change and its determinants. Given the limited research assessing the climate change literacy of adolescents on a nationwide scale, an essential consideration of Indonesia’s diverse population is crucial in further studies.

## 2. Materials and Methods

Data were collected from February to March in 2023. The validity and reliability of the questionnaire was first assessed with 41 adolescents from Java Island as respondents. The questionnaire consisted of close-ended questions and it included multiple options, one of which was the correct answer. The questionnaire was subjected to a factor analysis, and a loading factor of 0.4–0.84 was obtained, indicating that the items were valid and reliable. To ensure the feasibility, readability, consistency of style, and clarity of the language used, we conducted a face validity assessment through a simple interview involving a public health expert and 10 adolescents. We revised the questionnaire based on the input we obtained from the interview to ensure the clarity and relevance of the questionnaire. 

**Figure 1:**
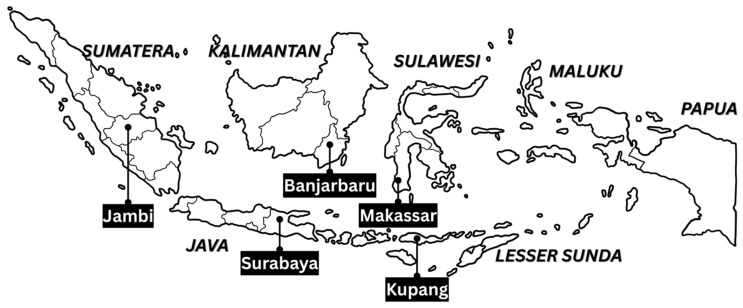
Map showing sampled regions in Indonesia.

The study population consisted of Indonesian adolescents aged 13–19 years old. To ensure a representative sample and to minimize bias, we used a multi-stage sampling approach:

- Stage One: The five major islands in Indonesia, namely, Sumatra, Java, Kalimantan, Sulawesi, and Eastern Indonesia, were selected through total enumeration sampling.
- Stage Two: In each major island, random sampling was used to choose one province and one city. The cities chosen were Jambi (Sumatra), Surabaya (Java), Banjarbaru (Kalimantan), Makassar (Sulawesi), and Kupang (Nusa-Papua). The location is shown in the Figure 1.
- Stage Three: In each province, two junior and two senior high schools were randomly selected using simple random sampling.
- Stage Four: In each school, purposive sampling was used to select 60 students, who were allowed to participate by their respective teachers, those being their guardians in school. This approach ensured homogeneity and representativeness in the schools being studied. The total number of respondents was 1126.

Data collection was guided by research ethics, wherein informed consent was first obtained from the students and their teachers. Survey Questionnaire of Climate Change among Indonesian Adolescents was used to collect data as an internet-based self-administered questionnaire (Supplementary File S1). To minimize response biases, we asked the students to fill out the questionnaire at school so that they could be supervised by their teachers and by the trained field team. During data collection, the field team thoroughly explained the guidelines to the students to ensure that they would follow the questionnaire’s instructions. In each school, the average questionnaire response rate was 93.3%.

Univariate analysis was used to determine the frequency distribution of the respondents’ characteristics and their answers to the knowledge assessment questions. The highest possible score was 100% (20 correct answers). Climate change literacy was categorized as follows: poor (score ≤ 55% or <11 correct answers), moderate (56–75% or 11–15 correct answers), and good (76–100% or 16–20 correct answers) [30]. A chi-square test was used to determine the relationship between the respondents’ characteristics and their knowledge. Multinomial logistic regression analysis was used because the dependent variable consisted of three knowledge categories. Multinomial logistic regression was used to determine the effect of each independent variable on the level of knowledge (moderate knowledge compared to poor and good knowledge compared to poor). The effect of each independent variable was measured by the odds ratio (OR). The OR in multiple logistic regression can be interpreted as the chance of a particular independent group to experience moderate or good knowledge compared to poor knowledge, after controlling for a set of other independent variables.

## 3. Results

This research involved 1126 respondents aged 13–19 years old. Table 1 presents the general characteristics of the respondents, who came from the five regions of Indonesia, namely, Java (20%), Sumatra (21.2%), Kalimantan (19.6%), Sulawesi (19.6%), and Eastern Indonesia (19.5%). Of the respondents, 60% were female, 49.8% were 8th grade students, 50.2% were 11th grade students, 34.9% were from private schools, 30.5% were from public schools, 19.6% were attending religious schools, and 15% were attending vocational schools. Most of the respondents had parents who attended high school (45.2%) and university (30.6%). The majority of the parents were entrepreneurs (28.4%) and private employees (23.2%).

The main sources of information used to gain climate change literacy were the internet (51.4%) and schools (40.9%). Table 1 shows that nearly half of the respondents have poor knowledge (49.7%), one third have moderate knowledge (32.4%), and only 17.9% have good knowledge.

Table 2 shows the knowledge items. Good knowledge was observed in the following aspects: climate extremes and fatality, climate change and quality of health in children and adolescents, the causes of climate change, climate change and vector-borne diseases, climate change, and communicable diseases. More than 70% of the respondents demonstrated knowledge about these concerns.

By contrast, poor knowledge was observed regarding the following categories: climate change and mental health, pro-environmental behavior, policies related to economy and climate change, the greenhouse effect, factors affecting global warming, transport-related policies and climate change, energy-related policies and climate change, and fossil fuels and climate change. Only 45.3% of the adolescents gave a correct answer regarding the greenhouse effect. Half of the respondents gave a wrong answer to the question on the causes of global warming (50.5%). More than half of the respondents were wrong about the most effective policies related to energy (64%), transportation (54.2%), and public protection (58%).

The chi-square test results shown in Table 3 show significant relationships for region (*p* < 0.001), age (*p* < 0.001), type of school (*p* = 0.006), grade (*p* < 0.001), parents’ education (*p* < 0.001), parents’ occupation (*p* < 0.001), and information source (*p* < 0.001) with adolescents’ knowledge regarding climate change.

Multinomial logistic regression analysis was conducted to assess the determinants of adolescents’ knowledge about climate change. Table 4 shows that the adolescents from Java (*p* < 0.001, OR = 4.7), Sumatra (*p* = 0.022, OR = 2.23), and Kalimantan (*p* < 0.001, OR = 4.2) have better knowledge (moderate and good) about climate change than those from Sulawesi and the eastern part of Indonesia. The adolescents studying in public school (*p* < 0.001, OR = 3.47) or religious school (*p* = 0.042, OR = 1.98) also demonstrated better (good) knowledge about climate change than those studying in private or vocational schools. Those in senior high school (*p* < 0.001, OR = 3.2) similarly showed better knowledge (moderate and good) than those in junior high school. The adolescents whose parents went to college (*p* < 0.001, OR = 3.4) or senior high school (*p* = 0.004, OR = 1.94) have higher climate change literacy (moderate and good) than the adolescents whose parents had less education. Higher climate change literacy was also observed among adolescents whose parents were private employees (*p* < 0.001, OR = 4.75), civil servants/army/police (*p* = 0.002, OR = 3.31), or entrepreneurs (*p* = 0.002, OR = 3.01) than among adolescents whose parents were farmers, retired, or unemployed. Students who use the internet as their source of information regarding climate change (*p* < 0.001, OR = 3.18) have better knowledge than students who use other sources to learn about the climate crisis.

## 4. Discussion

Climate change is a global issue that is one of the foci of the Sustainable Development Goals. Knowledge about climate change has become a necessity for all individuals worldwide in order to mitigate the impacts of climate change. However, adolescents, being one of the population segments affected by climate change, remain minimally involved in climate change campaigns. This study finds that nearly half of Indonesian adolescents have poor knowledge (49.7%) about climate change. This finding is in line with a study conducted in Bogor, Indonesia, which revealed that, out of 142 junior high school students, 30.99% had poor knowledge of climate change issues [23]. Sulistyawati et al. [25] conducted a study involving 508 high school students in Yogyakarta and found that students had low and inconsistent understandings of climate change and its impact on health [25]. Similar regional trends have been observed globally, where only 50% of young people have a correct understanding of climate change, with most well-informed individuals coming from upper-middle- and high-income countries [31]. A comparable trend is evident in the Asia–Pacific region, where the youth generally possess a low to moderate level of climate change knowledge [32]. Studies have shown that, compared with young people in other Southeast Asian countries, such as Malaysia, Singapore, the Philippines, and Thailand, young people in Indonesia have a moderate level of climate change literacy, and these studies recommend further improvements in the educational system [33,34,35,36]. Specifically, ISEAS [37] reported that only 79.3% of Indonesian answered correctly on a climate change survey. This number was lower than the overall Southeast Asian average of 82.3%, and Indonesia ranks among the lowest—7 out of 10—for countries in the Southeast Asia region [37].

Climate change knowledge is a big investment for future generations. The importance of knowledge about climate change and its correlation is based upon the Theory of Planned Behavior, which is a theory of attitudes and willingness to act in an environmental context [38]. This theory is widely used to interpret the influence of information dissemination on the development of personal attitudes. Human behavior is determined mainly by one’s intent to act, by social norms, and by a locus of control. Specific knowledge is a part of these three main determinants [39]. A study by Harmuningsih et al. [24] involving 120 Indonesians aged 15–35 years found that knowledge of climate change had a positive and significant effect on pro-environmental behavior intentions (*p*-value = 0.000) [24]. In addition, a literature review concluded that, in Indonesia, knowledge of climate change is fundamental to maintaining the well-being of adolescents [40]. The present study finds a significant relationship between region, age, type of school, class, parents’ education, and parents’ occupation with adolescents’ knowledge regarding climate change. This finding aligns with that of another study by Puspita et al. conducted in Bogor, Indonesia, wherein junior high school students’ knowledge was shown to be related to family background factors, such as household income and mother’s education [23].

Moreover, this study finds that adolescents from the western area (i.e., Surabaya, Jambi, and Banjarbaru) of Indonesia had better knowledge about climate change (moderate and good) than those from the eastern area (i.e., Makassar and Kupang). This result may be attributed to education inequality in Makassar and Kupang. The Ministry of National Development Planning reports that the Makassar area, which is part of Sulawesi Island, and Kupang, which is part of the Nusa Tenggara Islands, are categorized as underdeveloped areas [41].

Another finding is that adolescents from public and religious schools have better (good) knowledge about climate change than those from private and vocational schools. In Indonesia, the quality of education in vocational and private schools is often seen to be not as high as in public and religious schools. This notion is due to the more stiff competition one has to go through when entering public schools than when entering vocational and private schools [42]. Moreover, this study finds that adolescents with college-educated parents had better knowledge about climate change (moderate and good) than those with parents whose highest education attainment was elementary or secondary education. The results of this study are in line with those of an Ethiopia-based study, which showed a positive relationship between parents’ education and children’s academic achievement (r = 0.73). The results show that parents’ education is an important variable for predicting children’s academic achievement and knowledge [43].

The scores obtained by the adolescents with moderate knowledge relative to those of the adolescents with poor knowledge had an odds ratio (OR) of 1.66–3.02. Efforts to improve adolescents’ knowledge (from moderate to good) need to optimize the OR to 1.94–4.75. The present results indicate the urgent need to achieve equality in climate change literacy for disadvantaged adolescents in Indonesia. Climate change poses risks and threatens the health and well-being of adolescents. Adolescents are our future leaders and agents of change who will make decisions in the face of increasingly severe climate conditions. Policies related to climate change are important in protecting the adolescents of the present and of the future [11]. In fact, although a well-structured policy framework is in place in Indonesia, significant challenges prevent its implementation, enforcement, and the mobilization of adequate investments for sustainable practices [44,45]. Strengthening governance, enhancing sectoral coordination, and fostering multilevel collaboration, including in public education, are essential to overcoming these obstacles [45].

Regrettably, the Ministry of Education and Culture of Indonesia has not expressed support for the sixth development agenda, namely, developing the environment, increasing disaster resilience, and mitigating climate change [27]. The Regulation of the Ministry of Education, Culture, Research and Technology of the Republic of Indonesia Number 13 of 2022 regarding Amendments to the Regulation of the Minister of Education and Culture Number 22 of 2020 concerning the Strategic Plan of the Ministry of Education and Culture for 2020–2024 explains that the Ministry of Education and Culture will only provide support for the 2020–2024 development agenda, specifically the third (increasing quality and competitive human resources), fourth (mental revolution and cultural development), and seventh (strengthening the stability of politics, law, and security and transforming public services) agendas [27]. The Indonesian Government’s commitment and support are needed to increase the climate change literacy of its adolescents.

One study found that, in Indonesia, the education curriculum still inadequately supports climate change education due to teachers’ limited understanding and capacity to teach the topic. Additionally, the curriculum places excessive emphasis on cognitive knowledge while neglecting socio-emotional and behavioral learning aspects, highlighting the need for improvements in formal education, such as scouting or other extracurricular activities [46].

Another significant challenge affecting climate change education is the educational disparities across different regions in Indonesia, along with inequalities between vocational and private schools. These disparities underscore the importance of informal education as a complementary approach in enhancing climate change literacy among Indonesian adolescents, particularly through online platforms, such as social media. Our findings show that 51.4% of Indonesian adolescents primarily access climate change information online, and 40.9% rely on schools as their secondary source of information. A study conducted by the Association of Indonesian Internet Service Providers (APJII) in 2021, during which the COVID-19 pandemic hit the world, found that the internet penetration rate in Indonesia grew to 77.02% from 73.70% during the previous year [29]. They also found that adolescents were among the biggest contributors to this increase because schools in Indonesia held classes via distance learning. In addition, APJII found that, in 2021, adolescents aged 13–18 years old constituted the largest group of internet users in Indonesia [29].

Addressing regional disparities in the local context requires specific and locally rooted interventions, especially within Indonesia’s decentralized governance structure. An effective climate policy must reinforce and clarify the roles of subnational governments, making community-based and locally led approaches essential for inclusive local climate action [47]. For instance, credible and locally tailored climate change literacy campaigns in schools and online are crucial for increasing adolescents’ knowledge and awareness. Collaboration among the government, NGOs, and other stakeholders is essential in expanding these campaigns, particularly through social media and school-based programs. One example of utilizing media to enhance climate change education can be seen in The Gambia, wherein the national media produces three televised programs featuring phone-in segments on climate change and related issues in local languages [48]. Education that delivers knowledge about the environment and climate change, either through formal education in schools or informal education through another information dissemination from credible institutions, is important in equipping adolescents with the ability to defend their right to health and well-being, as well as to defend others’ rights in the future.

This endeavor ensures a sustainable positive behavioral change related to action for climate change among adolescents. In some schools in Indonesia, climate change education has been incorporated into geography subjects. However, public awareness campaigns, especially among adolescents, remain rare, except in schools located in major cities. These campaigns may be integrated into school activities, such as extracurricular programs. One good practice, seen in Brazil, is that efforts have been made to establish a research-based approach to mitigating climate change and disaster risk at the local level as part of outdoor school activities [49].

Regarding teacher training, scouting activities in middle and high schools are often supervised by teachers. Climate change may be proposed as a training topic for teachers to explore, as it has not yet been considered a significant subject in teacher training programs in Indonesia. Notably, the best practices in the Dominican Republic highlight substantial investments (USD 30,000) in climate change training for teachers, with a focus on enhancing their knowledge and pedagogical skills to effectively integrate climate education into the curriculum [50].

## 5. Conclusions

This study finds that 49.7% of Indonesian adolescents have poor knowledge regarding climate change. Their main sources of information about climate change are the internet (51.4%) and school subjects (40.9%). Adolescents’ knowledge on climate change was significantly related to their region: adolescents from Java (*p* < 0.001, OR = 4.7), Sumatra (*p* = 0.022, OR = 2.23), and Kalimantan (*p* < 0.001, OR = 4.2) had better knowledge (moderate and good) than those from Sulawesi and the eastern part of Indonesia. Adolescents studying in public (*p* < 0.001, OR = 3.47) or religious (*p* = 0.042, OR = 1.98) schools have better (good) knowledge about climate change than those studying in private or vocational schools. Senior high school students (*p* < 0.001, OR = 3.2) have higher climate change literacy (moderate and good) than junior high school students. Climate change literacy was also higher (moderate and good) among adolescents whose parents went to college (*p* < 0.001, OR = 3.4) or senior high school (*p* = 0.004, OR = 1.94) than those whose parents had less formal education. Adolescents whose parents are private employees (*p* < 0.001, OR = 4.75), civil servants/army/police (*p* = 0.002, OR = 3.31), or entrepreneurs (*p* = 0.002, OR = 3.01) also have better knowledge than students whose parents are farmers, retired, or unemployed. Students with the internet as their source of information regarding climate change (*p* < 0.001, OR = 3.18) have better knowledge than students who learn about this topic from other sources. More comprehensive and collaborative efforts across sectors are needed to effectively enhance adolescents’ knowledge about climate change. This requires high-quality formal education, which involves well-trained teachers with expertise in climate change and a structured, age-appropriate curriculum. Additionally, informal media, such as media training and social media-based movements, can help broaden the outreach. These efforts should be reinforced through strong policy actions that align with Indonesia’s decentralized governance system. Furthermore, educational materials and curricula should be adapted to local and cultural contexts to ensure long-term, sustainable behavioral change arising from climate crisis awareness.

## Figures and Tables

**Table 1:** Characteristics of adolescents aged 13–19 years from five cities in Indonesia.

Variable	Frequency (*n* = 1126)	
	*n*	%
1. Region		
Surabaya	225	20.0
Jambi	239	21.2
Banjarbaru	221	19.6
Makassar	221	19.6
Kupang	220	19.5
2. Age (years)		
13	183	16.3
14	290	25.8
15	100	8.9
16	215	19.1
17	212	18.8
18	114	10.1
19	12	1.1
3. Sex		
Female	676	60.0
Male	450	40.0
4. Type of School		
Public	343	30.5
Religious (Muslim/Christian School/Catholic School)	221	19.6
Vocational	169	15.0
Private (International and Local)	393	34.9
5. Grade		
8 (Junior High School)	561	49.8
11 (Senior High School)	565	50.2
6. Parents’ Education		
Elementary School	74	6.6
Junior High School	184	16.3
Senior High School	509	45.2
University	345	30.6
Never Attended School	14	1.2
7. Parents’ Occupation		
Civil Servant/Army/Police	166	14.7
Private Employee	261	23.2
Entrepreneur	320	28.4
Farmer	85	7.5
Retired	18	1.6
Unemployed	38	3.4
Others	238	21.1
8. Knowledge About Climate Change		
Poor (≤55)	560	49.7
Moderate (56–74)	365	32.4
Good (≥75)	201	17.9

**Table 2:** Indonesian adolescents’ knowledge regarding climate change (n = 1126).

Climate Change Aspects	Correct (%)	Wrong (%)	Do Not Know (%)
Climate extremes (such as floods, landslides, forest fires and heat waves) can cause death	87.7	6.8	5.5
Climate change affects the quality of health of children and adolescents	84.0	6.3	9.7
Climate change is primarily caused by humans	79.0	12.5	8.5
The incidence of infectious diseases, such as hemorrhagic fever (DHF), increases due to climate change	74.9	12.0	13.1
Climate change can increase the incidence of foodborne and waterborne diseases such as diarrhea	71.0	12.7	16.3
Climate change affects one’s mental health (e.g., it causes stress)	65.4	18.7	15.9
What does pro-environmental behavior mean?	62.9	30.4	6.7
What is the meaning of the greenhouse effect?	45.3	44.0	10.7
What contributes most to global warming?	43.5	50.5	6.0
What are the most effective measures to address the climate crisis?			
1. Economy-related policies	61.1	29.1	9.8
2. Transportation-related policies	36.3	54.2	9.5
3. Community protection	31.9	58.0	10.1
4. Energy-related policies	28.8	64.0	7.2
5. If we stop burning fossil fuels, the level of carbon dioxide in the atmosphere will immediately decrease	10.0	71.3	18.7
Which of the following is a fossil fuel?			
1. Oil	77.1	14.3	8.6
2. Coal	72.4	18.2	9.4
3. Natural gas	70	16.9	13.1
4. Wood	53.8	27.2	19
5. Hydrogen	43.1	32.5	24.4
6. Solar	42.5	38.1	19.4

**Table 3:** Chi-Square analysis of Indonesian adolescents’ knowledge regarding climate change.

Variables		Knowledge Category *n* (%)			Total (*n*)	*p*-Value
		Poor (≤55%)	Moderate (56–74%)	Good (≥75%)		
Region	Java	89 (39.6%)	77 (34.2%)	59 (26.2%)	225	<0.001
	Sumatra	111 (46.4%)	79 (33.1%)	49 (20.5%)	239	
	Kalimantan	78 (35.3%)	98 (44.3%)	45 (20.4%)	221	
	Sulawesi	142 (64.3%)	53 (24%)	26 (11.8%)	221	
	Eastern Indonesia	140 (63.6%)	58 (26.4%)	22 (10%)	220	
Age (years)	13–15	339 (59.2%)	173 (30.2%)	61 (10.6%)	573	<0.001
	16–17	157 (36.8%)	155 (36.3%)	115 (26.9%)	427	
	18–19	64 (50.8%)	37 (29.4%)	25 (19.8%)	126	
Sex	Female	318 (47%)	230 (34%)	128 (18.9%)	676	0.085
	Male	242 (53.8%)	135 (30%)	73 (16.2%)	450	
Type of School	Public	150 (43.7%)	113 (32.9%)	80 (23.3%)	343	0.006
	Religious (Muslim/Christian School/Catholic School)	114 (51.6%)	71 (32.1%)	36 (16.3%)	221	
	Vocational	76 (45.0%)	61 (36.1%)	32 (18.9%)	169	
	Private (International and Local)	220 (56.0%)	120 (30.5%)	53 (13.5%)	393	
Grade	8 (Junior High School)	330 (58.8%)	169 (30.1%)	62 (11.1%)	561	<0.001
	11 (Senior High School)	230 (40.7%)	196 (34.7%)	139 (24.6%)	565	
Parents’ Education	Never Attended School or Elementary School	54 (61.4%)	24 (27.3%)	10 (11.4%)	88	<0.001
	Junior High School	121 (65.8%)	42 (22.8%)	21 (11.4%)	184	
	High school	244 (47.9%)	181 (35.6%)	84 (16.5%)	509	
	University	141 (40.9%)	118 (34.2%)	86 (24.9%)	345	
Parents’ Occupation	Civil Servant/Army/Police	84 (50.6%)	48 (28.9%)	34 (20.5%)	166	<0.001
	Private Employee	112 (42.9%)	84 (32.2%)	65 (24.9%)	261	
	Entrepreneur	144 (45.0%)	123 (38.4%)	53 (16.6%)	320	
	Farmer	54 (63.5%)	25 (29.4%)	6 (7.1%)	85	
	Retired	8 (44.4%)	7 (38.9%)	3 (16.7%)	18	
	Unemployed	28 (73.7%)	8 (21.1%)	2 (5.3%)	38	
	Others	130 (54.6%)	70 (29.4%)	38 (16.0%)	238	
Information Source	Internet (Social Media and Other Websites)	241 (41.6%)	208 (35.9%)	130 (22.5%)	579	<0.001
	School	260 (56.5%)	139 (30.2%)	61 (13.3%)	460	
	Others	59 (67.8%)	18 (20.7%)	10 (11.5%)	87	

**Table 4:** Multiple logistic regression models that predict good and moderate knowledge about climate change among Indonesian adolescents *.

Variables		Knowledge Category							
		Moderate (56–74)				Good (≥75)			
		*p*-Value	OR	95% CI (B)		*p*-Value	OR	95% CI (B)	
				Lower	Upper			Lower	Upper
Region	Java	0.001	2.44	1.46	4.07	<0.001	4.70	2.41	9.16
	Sumatra	0.009	1.99	1.18	3.34	0.022	2.23	1.12	4.42
	Kalimantan	<0.001	3.02	1.87	4.89	<0.001	4.19	2.12	8.28
	Sulawesi	0.695	1.11	0.65	1.92	0.101	1.86	0.89	3.92
	Eastern Indonesia (Reff)		1.0				1.0		
Age (years)	13–15	0.927	0.96	0.39	2.38	0.097	0.32	0.08	1.23
	16–17	0.379	1.27	0.75	2.14	0.586	1.19	0.64	2.22
	18–19		1.0				1.0		
Type of School	Public	0.059	1.47	0.99	2.20	<0.001	3.47	2.01	6.00
	Religious	0.164	1.43	0.86	2.35	0.042	1.98	1.02	3.83
	Vocational	0.817	0.94	0.57	1.56	0.949	1.02	0.55	1.90
	Private		1.0				1.0		
Grade	11 (Senior High School)	<0.001	1.664	1.276	2.171	<0.001	3.22	2.28	4.53
	8 (Junior High School)		1.0				1.0		
Parents’ Education	University	<0.001	2.219	1.526	3.226	<0.001	3.44	2.16	5.49
	Senior High School	<0.001	1.967	1.397	2.770	0.004	1.94	1.23	3.06
	Never Attended School, Elementary School, and Junior High School		1.0				1.0		
Parents’ Occupation	Private Employees	0.028	1.688	1.057	2.694	<0.001	4.75	2.37	9.53
	Civil Servant/Army/Police	0.338	1.286	0.769	2.150	0.002	3.31	1.58	6.96
	Entrepreneur	0.004	1.922	1.234	2.994	0.002	3.01	1.49	6.07
	Others	0.426	1.212	0.755	1.943	0.018	2.39	1.16	4.93
	Farmer, Retired, and Unemployed		1.0				1.0		
Information Source	Internet (Social Media and Other Websites)	<0.001	2.829	1.617	4.950	0.001	3.18	1.57	6.43
	School	0.052	1.752	0.994	3.088	0.380	1.34	0.67	2.86
	Others		1.0				1.0		

* Reference category of dependent variable is poor knowledge (≤55).

## Data Availability

The raw data supporting the conclusions of this article will be made available by the authors upon request. As stated in the informed consent form, data obtained from the respondents will not be publicly available so as to protect the respondents’ rights to data security.

## Supplementary Materials

The following supporting information can be downloaded at: https://www.mdpi.com/article/10.3390/ijerph22040571/s1, File S1: Survey Questionnaire of Climate Change among Indonesian Adolescents.
