# Supplementary material for: Adolescents’ Knowledge on Climate Change: A Nationwide Study in Indonesia
Source: Int J Environ Res Public Health. 2025 Apr 5;22(4):571. doi: 10.3390/ijerph22040571 (PMC12026836; doi:10.3390/ijerph22040571)
Supplement: Supplementary file 1 [file ijerph-22-00571-s001.zip › ijerph-3495805-supplementary.pdf]

Supplementary File S1. The questionnaire of knowledge about climate change among Indonesian adolescents

Questions	Options
Climate extremes (such as floods, landslides, forest fires and heat waves) can cause death	**a. Yes**<br>b. No<br>c. Do not know
Climate change affecting the quality of health of children and adolescents	**a. Yes**<br>b. No<br>c. Do not know
Climate change is primarily caused by humans	**a. Yes**<br>b. No<br>c. Do not know
The incidence of infectious diseases, such as hemorrhagic fever (DHF), increases due to climate change	**a. Yes**<br>b. No<br>c. Do not know
Climate change can increase the incidence of foodborne and waterborne diseases such as diarrhea	**a. Yes**<br>b. No<br>c. Do not know
Climate change affects one's mental health (e.g., it causes stress)	**a. Yes**<br>b. No<br>c. Do not know
What does pro-environmental behavior mean?	**a. Conscious behavior is used to minimize the negative impact of our actions on nature**<br>b. Unconscious behavior to minimize the negative impact of our actions on nature<br>c. Behavior to minimize the positive impact of our actions on nature<br>d. Behavior to maximize the negative impact of our actions on nature<br>e. Do not know
What is the meaning of the greenhouse effect?	**a. Gas in the atmosphere that holds hot air out of the Earth**<br>b. The earth's protective ozone layer is damaged<br>c. Pollution that causes acid rain to occur<br>d. How plants grow and develop<br>e. Do not know
What contributes most to global warming?	a. Cars and trucks<br>**b. Burning fossil fuels for heating and electricity**<br>c. Holes in the ozone layer<br>d. Deforestation<br>e. Do not know

**What is the most effective measures to address the climate crisis?**	
1. Economy-related policies	**a. Investing more in green businesses that balance profit and environmental sustainability**<br>b. Requiring the provision of complete information about the manufacture of a product<br>c. Expanding the industrial sector<br>d. Making companies pay for the pollution they produce<br>e. Do not know
2. Transportation-related policies	a. Use more electric cars, motorcycles, and buses<br>**b. Use renewable energy for transport**<br>c. Improve urban and rural design<br>d. Provide adequate space for pedestrians

	e. Do not know
3. Community protection	a. Coordinate disaster agencies<br>b. Install more early warning systems for disasters<br>c. Provide good and affordable insurance<br>**d. Build infrastructure and nature conservation to protect living things and their habitats**<br>e. Do not know
4. Energy-related policies	**a. Using solar, wind, and other renewable energy**<br>b. Conserving energy use in homes, buildings, and buildings<br>c. Stopping the burning of polluting fuels<br>d. Building coal-fired power plants<br>e. Do not know
5. If we stop burning fossil fuels, the level of carbon dioxide in the atmosphere will immediately decrease	a. Yes<br>**b. No**<br>c. Do not know
**Which of the following is a fossil fuel?**	
1. Oil	**a. Yes**<br>b. No<br>c. Do not know
2. Coal	**a. Yes**<br>b. No<br>c. Do not know
3. Natural gas	**a. Yes**<br>b. No<br>c. Do not know
4. Wood	a. Yes<br>**b. No**<br>c. Do not know
5. Hydrogen	a. Yes<br>**b. No**<br>c. Do not know
6. Solar Energy	a. Yes<br>**b. No**<br>c. Do not know

Scoring:
Correct answer (bold format) = 1
Do not know and other false answers = 0
